# Method to increase the yield of eukaryotic membrane protein expression in *Saccharomyces cerevisiae* for structural and functional studies

**DOI:** 10.1002/pro.2507

**Published:** 2014-06-20

**Authors:** Joanne L Parker, Simon Newstead

**Affiliations:** Department of Biochemistry, University of OxfordSouth Parks Road, Oxford, OX1 3QU, United Kingdom

**Keywords:** eukaryotic membrane protein expression, green fluorescent protein, structural-functional analysis, *Saccharomyces cerevisisae*

## Abstract

Despite recent successes in the structure determination of eukaryotic membrane proteins, the total number of structures of these important proteins is severely underrepresented in the Protein Data Bank. Although prokaryotic homologues provide valuable mechanistic insight, they often lack crucial details, such as post-translational modification and additional intra or extracellular domains that are important for understanding the function and regulation of these proteins in eukaryotic cells. The production of milligram quantities of recombinant protein is still a serious obstacle to the structural and functional characterization of these proteins. Here, we report a modification to a previously described over expression system using the simple eukaryote *Saccharomyces cerevisiae* that can increase overall protein yield and improve downstream purification procedures. Using a metabolic marker under the control of a truncated promoter, we show that expression levels for several membrane transporters are increased fourfold. We further demonstrate that the increase in expression for our test proteins resulted in a concomitant increase in functional protein. Using this system, we were able to increase the expression level of a plant transporter, NRT1.1, which was a key factor in its structural and functional characterization.

## Introduction

Substantial progress has recently been made tackling the many challenges associated with determining the crystal structures of membrane proteins (MPs; reviewed in Ref.^1^). These have included novel methods for screening detergents,^2^ engineering proteins for increased thermal stability,^3^ optimizing crystallization conditions,^4^ crystallization methodology,^5^ and the collection of the diffraction data using microfocus beamlines.^6^ These developments among many others have lead to a substantial increase in the number of MP structures that have been determined over the past decade. However, the number of structures is still small compared to soluble proteins and even more pronounced is the dominance of prokaryotic structures that still vastly outnumber eukaryotic ones.^7^ For many, the main stumbling block to working on eukaryotic MPs is over expression and purification at a level that will yield milligram amounts of pure, functional, and homogenous protein for downstream structural and biophysical studies at a reasonable cost.

With the difficulties associated with crystallizing MPs many groups have opted for medium throughput screening procedures to identify those targets that appear more amenable to structural studies.^8^ These technologies often utilize green fluorescent protein (GFP) tagging of the proteins of interest to follow their expression, solubilization efficiency, and monodispersity through fluorescent size exclusion chromatography (FSEC).^9^ This strategy has been successfully applied in *Escherichia coli*,^10^ *Lactococcus lactis*,^11^ *Saccharomyces cerevsiaie*,^12^ and in *Pichia pastoris*.^13^ However, even with these improved screening systems in place to identify potentially suitable target proteins, the problem of moving to a large scale production system for their over expression is still far from being a trivial scaling problem.

Eukaryotic MPs tend to require a eukaryotic expression system for their overproduction.^14^,^15^ It is thought that this requirement stems from optimized interactions with the Sec translocon and post-translational modifications, such as correct glycosylation, which is sometimes essential for the production of functional protein.^16^ Reviewing the number of current eukaryotic MP structures determined and their associated over expression host reveals that to date baculovirus expression is proving the most successful method (43%). However, most of these structures come from recombinantly produced G-protein coupled receptors (GPCRs)^17^ (80%), followed by *P. pastoris* (19%), *Saccharomyces cerevisiae* (9%), and finally mammalian cells (4%). However, when looking at the number of different types of MP that have been crystallized from protein produced by these systems, it is clear that yeasts are proving the current front runner for diversity. Yeasts are simple eukaryotes that combine the ease and cost effectiveness of *E. coli* but come with the added benefit of eukaryotic folding pathways. *S. cerevsiaie* and *P. pastoris* and to a limited extend *S. pombe* have all been used to produce protein which has been successful for structural determination through crystallography.

However, as a means for recombinant protein production *S. cerevisiae* does have a few advantages over *P. pastoris*; the later have a very tough cell wall which can make lysis difficult and also rely on the use of integrated vectors unlike *Saccharomyces*, which has a well characterized and stable multicopy plasmid system; the 2 µm plasmid which has copy numbers from 10–40 copies per cell and is easily transformed.^18^ These factors have led many smaller groups to adopt *Saccharomyces* as a route to recombinant eukaryotic MP production.

Previously a system for the recombinant production of eukaryotic MPs using auxotrophic *S. cerevisiae* strains was reported^12^ that utilized a 2 µm plasmid containing a C-terminal GFP protein for the fluorescent-based monitoring of expression, detergent suitability, and purification.^19^ This system relied on a *URA3* selectable marker and was effective in the production of a range of alpha helical MPs from different structural families. Although some optimization of expression is possible using chemical chaperones and different promoters, we recently encountered a situation where more extensive optimization of the system was required to increase the expression of a plant transporter gene, NRT1.1 from *Arabidopsis thaliana*.^20^

Here, we report our modification to the *S. cerevisiae* over expression system that can substantially improve the level of recombinant protein produced and enhance the usefulness as a large-scale method for structural, functional, and biophysical studies of eukaryotic MPs. Through the use of a truncated promoter controlling the expression of the selective marker for the plasmid, we show that expression can be improved up to fourfold per cell compared with the previous system for a range of polytopic MPs. We also show that the increase in protein level did not adversely affect the quality of the protein as judged by both FSEC, purification, and functional reconstitution of the transporters into liposome systems for uptake assays.

## Results and Discussion

**Figure 1:**
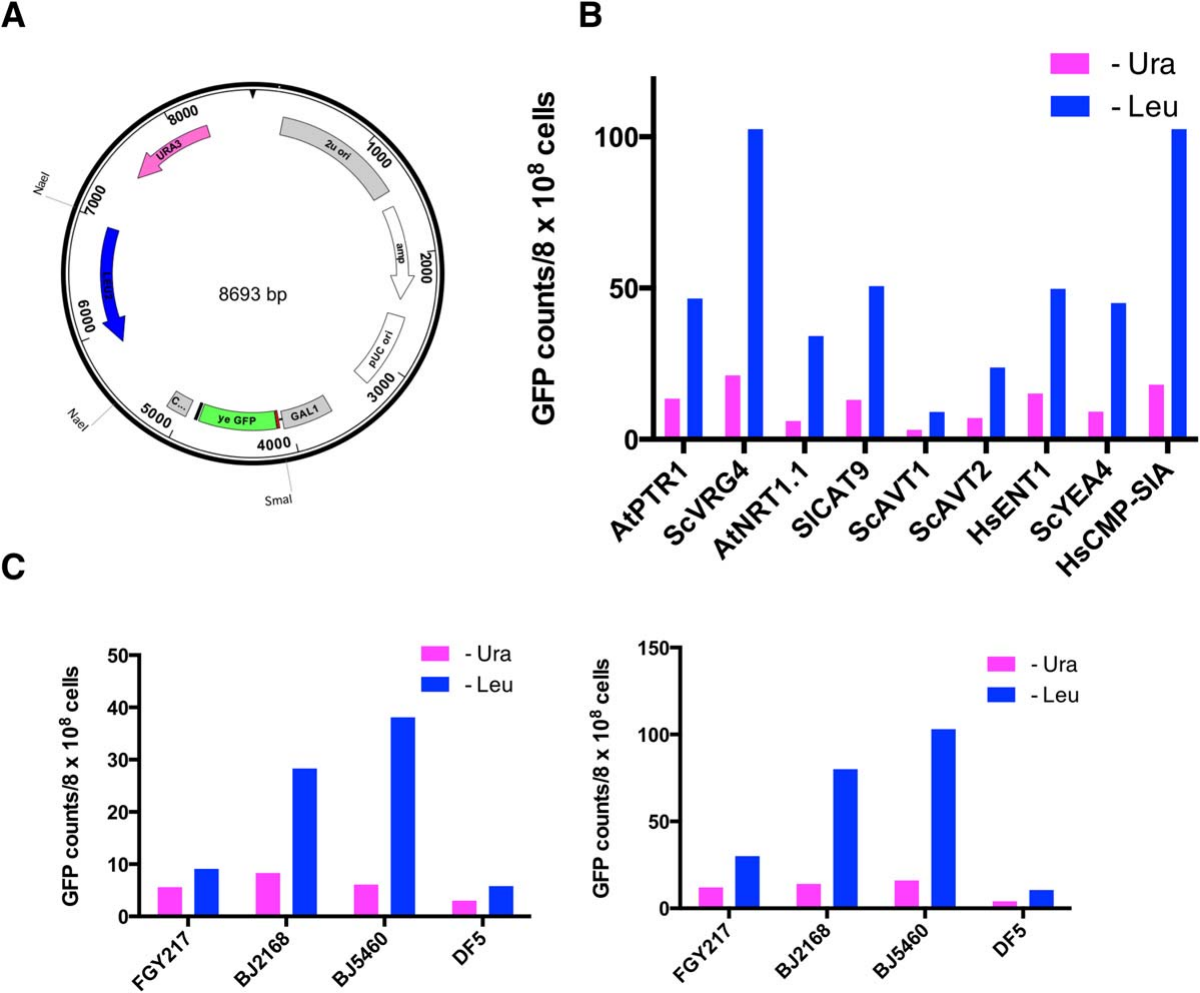
A modified *S. cerevisiae* expression system for eukaryotic MP production. (A) Vector map of pDDGFP2-Leu2d vector. The vector allows for dual selection using either the uracil or leucine markers. The gene for the recombinant protein is inserted via homologous recombination into the smaI site and is, then, expressed under the control of the GAL1 promoter as a tev cleavable C terminal tagged GFP-His fusion. (B) Comparison of expression level between the use of either the URA3 (selection using medium lacking uracil) or LEU2 genes (selection using medium lacking leucine) as selective markers for a number of different membrane proteins (see Table I for details)**.** (C) As (B) but looking at yeast strain dependence of the system for the expression of two constructs.

To improve the yield of recombinant integral MPs produced in *S. cerevsiae*, we introduced a *LEU2* gene containing a truncated version of its own promoter into the pDDGFP2 vector^12^ at the unique NaeI site [Fig. 1(A)]. The pDDGFP2 vector was chosen as it was used previously to over express a number of eukaryotic MPs.^12^ The pDDGFP2 vector contains the strong and inducible *GAL1* promoter, which is used to drive expression of the gene of interest, following addition of galactose as the carbon source during culturing. The recombinant MP is produced as a C-terminal yeast enhanced GFP fusion containing an octa-histadine affinity purification tag, this tag can be removed due to the presence of a Tobacco Etch Virus (TEV) protease site upstream of the GFP.^19^ The pDDGFP2 vector also allows for ligation free cloning through the use of regions flanking a unique SmaI site, which can be used to insert PCR products via homologous recombination through cotransformation of both PCR and SmaI lineralized vector into chemically competent yeast cells.

**Figure 2:**
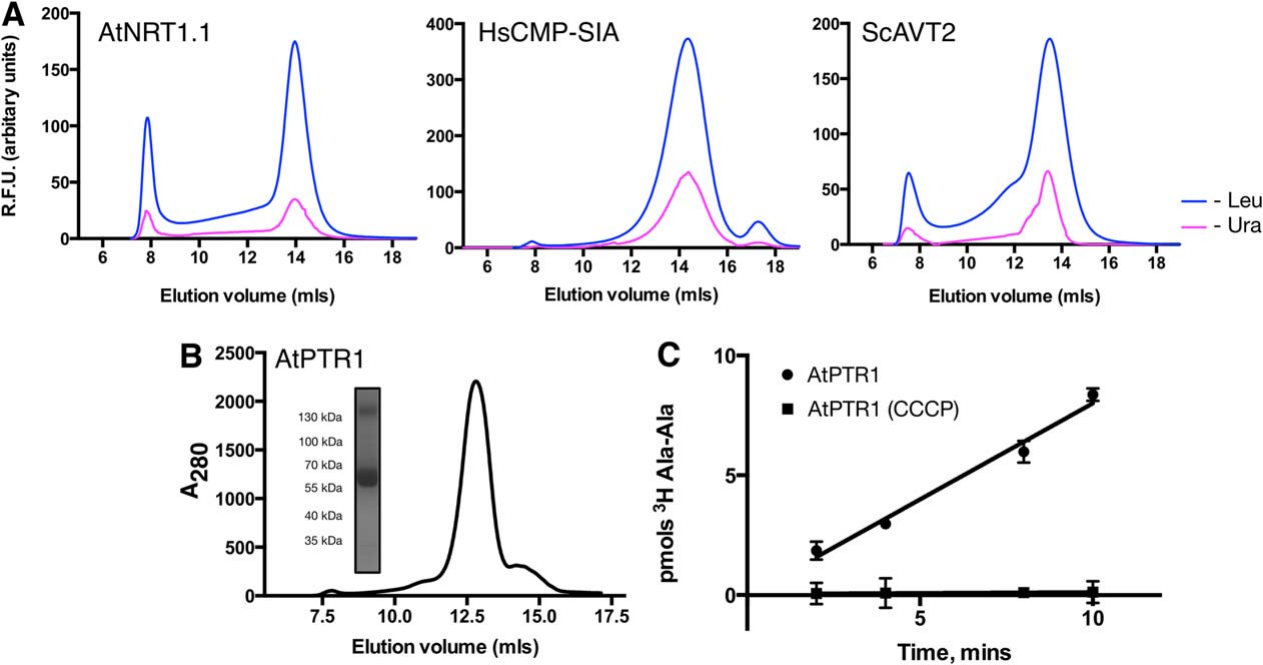
Analysis of the quality of the protein. (A) Representative FSEC analysis of three of the constructs obtained under both—uracil and—leucine media. (B) Gel filtration profile and SDS PAGE gel (inset) of the final step of purification of AtPTR1, please note that AtPTR1 runs as both a monomer and dimer on SDS PAGE but is monomeric by gel filtration. (C) Liposomes containing AtPTR1 can uptake tritiated di-alanine and this is proton dependent as the addition of the proton ionophore, CCCP, results in no uptake.

The newly constructed vector pDDGFP-Leu2d, then, allows for dual selection using either the *URA3* or the *LEU2D* markers. Due to the shortening and, hence, weakening of the promoter for the LEU2 gene, for the yeast to grow under the selective pressure of minus leucine medium the pDDGFP-Leu2d plasmid has to be maintained at a high copy number; 80–100 copies per cell compared to approximately 20 for the *URA3* marker. This increase in copy number can in turn lead to an increase in protein level.^21^ Nine different MPs, encompassing five different transporter families and four different eukaryotic organisms (both unicellular and multicellular) were screened (Table I) and in all cases a clear increase of between 2.9 and 5.7 times more fluorescence was measured from cells grown under minus leucine (−Leu) conditions compared to minus uracil (−Ura) for all proteins tested [Fig. 1(B)]. In addition, different yeast backgrounds were screened and in most cases again an increase in fluorescence was observed for −Leu conditions when compared to −Ura. The smallest difference was seen for the DF5 background where, under both selection conditions, only poor expression was seen [Fig. 1(C)]. To ensure the protein was of good quality and the increased expression level did not have a detrimental affect on the protein (i.e., aggregation or insoluble material), FSEC analysis was performed. In all cases, the constructs produced monodisperse traces, indicating that the increased expression did not lead to increased aggregation over expression in the –Ura medium [representative traces are shown in Fig. 2(A)]. Numerous targets have been produced and purified in milligram amounts from this system, for brevity here we show the purification [Fig. 2(B)] and functional characterization [Fig. 2(C)] of AtPTR1 a proton coupled peptide transporter from *A. thaliana*. Using this system, the final yield of purified, monodisperse, and functional AtPTR1 increased from 0.2 to 0.8 mg L^−1^.

**Table I:** Information on the Proteins used to Test the Leu2d System

Protein abbreviation	Protein name	Uniprot identifier	Predicted TM helices	Mass (KDa)
AtPTR1	*Arabidopsis thaliana* PTR1	Q9M390	12	64
ScVRG4	*Saccharomyces cerevisiae* VRG4	P40107	10	37
AtNRT1.1	*A. thaliana* NRT1.1	Q05085	12	64.9
SlCAT9	*Solanum lycopersicum* CAT9	K4CYY3	13	60.4
ScAVT1	*S. cerevisiae* AVT1	P47082	11	65.3
ScAVT2	*S. cerevisiae* AVT2	P39981	11	53.3
HsENT1	*Homo sapien* ENT1	Q99808	11	50.2
ScYEA4	*S. cerevisiae* YEA4	P40004	8	39.3
HsCMP-SIA	*H. sapien* CMP-SIA	P78382	8	36.8

In summary, we report the use of a truncated promoter for a metabolic marker of leucine, *Leu2d* to systematically increase the expression level per cell of nine target proteins. The increase in expression level is also coupled with a noted reduction in the overall OD of the culture following induction. In the previously reported system using the *URA* marker, final OD values were normally in the range of 10–12, equating to ∼2 × 10^8^ cells/mL. However, in the *Leu2d* system final OD values of 3–6 are more common; no doubt a direct result of the increased pressure of trying to grow with a truncated promoter. The benefit of this reduced cell density is that lysis efficiency can be increased using the same volumes as with the previous system and during purification the fraction of target protein to contaminants is massively increased, often resulting in improved purification on nickel affinity columns. The system presented here has been tested on over 20 eukaryotic integral MPs and resulted in increased yields over the same expression in the –Ura media. The purified proteins are functional and monodisperse and produced in amounts required for downstream structural and functional studies. We recently determined the crystal structure for one of the targets, NRT1.1 from *A. thaliana* that was made considerably more tractable through the improved expression obtained using this system.^20^ The increase in yield and ease of use in adapting existing laboratory infrastructure to culture *S. cerevisiae* make this system a valuable, cost effective route for the production of eukaryotic MPs for biophysical and biochemical studies.

## Materials and Methods

### Construction of the LEU2d vector and test vectors

The *LEU2* gene plus the *LEU2D* promoter was introduced by PCR and restriction digest into the unique NaeI site in the vector pDDGFP2 to create the new vector pDDGFP2-LEU2d. Target genes were amplified by PCR using gene specific primers including the homologous recombination regions for integration into this vector as previously described.^12^ The PCR products and *SmaI* digested vector were cotransformed into *S. cerevisiae* strain BJ5460 (MATa ura3-52 trp1 lys2-801 leu2Δ1 his3Δ200 pep4::HIS3 prb1Δ1.6R can1 GAL) and plated onto plates lacking uracil and left for 2 days at 30°C. The plasmid was recovered from the yeast and transformed into *E. coli* OMNIMAX cells (Invitrogen) and plated out onto LB agar plates containing 50 ug mL^−1^ ampicillin. From an individual *E. coli* colony, the plasmid containing the gene of interest was recovered using standard mini prep protocols (Qiagen); this was done to ensure a homogeneous plasmid stock for use in the expression screening. Plasmid concentrations were typically 350 ng uL^−^^1^. The pDDGFP2-LEU2d plasmid has been deposited in the Addgene (http://www.addgene.org) collection (#58352) for general dissemination.

### Strains

Four different *S. cerevisiae* strains were used to screen for expression of the recombinant protein. These were FGY217 (*MATa*, *ura3-52*, *lys2*_*201*, and *pep4*), BJ2168 (MATa prc1–407 prb1-1122 pep4-3 leu2 trp1 ura3-52), BJ5460, and DF5 (his3-Δ200, leu2-3,2-112, lys2-801, trp1-1(am), ura3-52). All studied strains were haploid. Plasmid transformation was carried out using the Lithium acetate method.^22^

### Plasmid construction

All genes were amplified using gene specific primers from either genomic DNA (for yeast genes) or their respective cDNA from commercial IMAGE clones. The cDNA for SlCAT9 was kindly provided by Professor Andrew Smith, Dept. of Plant Sciences, University of Oxford, Oxford, UK. Primers contained 30-bp homologous region on the forward and reverse primers for ligation independent cloning into the SmaI lineralized vector.

### Expression screening

The plasmid stock was transformed into the yeast strains and plated onto selective media lacking uracil. Due to the truncated promoter associated with the *LEU2* gene, after transformation with the plasmid the copy number of the plasmid has to increase to allow for growth on –Leu media. This can be achieved by first plating onto −Ura plates and then restreaking onto –Leu plates or after transformation the cells can be grown in YPD over night; both methods lead to an increase in copy number after the initial transformation and then survival on −Leu containing media.

To compare protein level in the two media types, overnight cultures were grown in liquid media with shaking at 250 rpm in 2% glucose in both of the selection media. This was diluted tenfold into 50 mL of selection media containing 2% lactate (pH 5.5) for 8 h, to allow for the derepression of the galactose promoter brought about by growth in glucose. Fusion protein expression was induced through the addition of 2% galactose for 20 h. The OD_600_ of each culture was tested and the equivalent of 8 × 10^8^ cells harvested, washed once in phosphate buffered saline (PBS) and then resuspended in 150 uL of PBS and transferred to a 96 well plate for measuring GFP fluorescence (excitation *λ* = 485 nm, emission *λ* = 512 nm) on a spectraMax M3 reader (Molecular Devices).

### FSEC analysis

Cells were harvested (4 × 10^9^) and washed once in PBS. The pellet was resuspended in 2 mL of PBS and lysed through the addition of glass beads and vortexing for 12 min at 4°C. Un-lysed material was removed through centrifugation at 12,000*g* and the membranes harvested by centrifugation for 1 h at 30,000*g* using a high-speed refrigerated microfuge (Eppendorf, GmbH). Membranes were resuspended in 0.5 mL PBS and stored at −80°C until required. Analysis of detergent solubilization efficiency and monodispersity was carried out using the standard FSEC procedure as detailed previously using a Superose 6 column (GE).^19^

### Large-scale cultures

Large-scale cultures were grown using 2.5 L Tunair flasks (VWR) containing 800 mL of –Leu media (6.7g L^−1^ YNB –Difco) 2% lactate. Starter cultures were grown in 100 mL of –Leu media in 500 mL baffled Erlenmeyer flasks for 20 h at 220 rpm at 30°C before being used to inoculate the 800 mL media. The flasks were then grown for a further 24 h at 250 rpm, 30°C before the addition of 2% galactose to induce expression of the recombinant genes. Expression was monitored using whole cell fluorescence and following maximal expression, anywhere from 6–12 h post induction, the cells were harvested at 10,000*g*, the pellet weighed and suspended at 5 mL PBS g^−1^.

### Purification

Cells were lysed using a constant system cell disruptor at 40,000 PSI (Constant Systems, UK). Cell debris was removed by centrifugation at 30,000*g* for 20 min and membrane isolated by ultracentrifugation and stored at −80 in PBS. Membranes were thawed and solubilized through the addition of 1% DDM for 1 h, insoluble material was removed through ultracentrifugation for 1 h at 200,000*g*. The recombinant fusion proteins were, then, purified by nickel affinity chromatography using nickel sepharose beads as previously described.^19^ The GFP tag was removed through protease cleavage using a ^His^TEV protease and both GFP and TEV protease were subsequently removed by running back through a HisTRap column (GE). The cleaved MP was concentrated to 0.5 mL and applied to a Superdex 200 size exclusion column.

### Reconstitution and transport Assay

AtPTR1 was reconstituted into soy lipids (Avanti Polar lipids) using the biobead method at a lipid:protein ratio of 60:1.^20^ For proton driven peptide uptake assays, artificially imposed potassium ion diffusion potentials were generated as previously described.^23^ Proteoliposomes were harvested at 90,000*g* for 30 min at 4°C and resuspended in 20 m*M* potassium phosphate, pH 6.50, 100 m*M* potassium acetate, 2 m*M* magnesium sulphate, followed by 11 cycles of extrusion through a 0.2 µm polycarbonate filter. Proteoliposomes were subsequently diluted 1:50 (v/v) to final protein concentration of 0.1 µ*M* into external buffer containing 20 m*M* sodium phosphate, pH 6.5, 2 m*M* magnesium sulphate with 10 m*M* valinomycin and ^3^H-labelled di-Ala peptide (50 µ*M*). Uptake of ^3^H substrate was assayed at 25°C. Reactions were stopped by diluting into 1.5 mL of ice-cold 0.1 *M* lithium chloride. Proteoliposomes were collected on 0.22 µm nitrocellulose filters and washed twice under vacuum with 0.1 *M* lithium chloride. The ^3^H signal was converted to pmol amounts using a standard curve of the substrate.
